# Prevalence of Electronic Cigarette Use Among Adult Workers — United States, 2017–2018

**DOI:** 10.15585/mmwr.mm7009a1

**Published:** 2021-03-05

**Authors:** Girija Syamlal, Kathleen A. Clark, David J. Blackley, Brian A. King

**Affiliations:** ^1^Respiratory Health Division, National Institute for Occupational Safety and Health, CDC; ^2^Office on Smoking and Health, National Center for Chronic Disease Prevention and Health Promotion, CDC.

Electronic cigarettes (e-cigarettes) heat a liquid to produce an aerosol that usually contains nicotine, flavors, and other chemicals and that is inhaled by the user (*1*). E-cigarette aerosols generally have a lower number and level of harmful toxicants than conventional cigarettes; however, e-cigarette aerosols can contain harmful ingredients, including ultrafine particles, volatile organic compounds, and heavy metals (*1*,*2*). The U.S. Surgeon General has determined that evidence is inadequate to conclude that use of e-cigarettes, in general, increases smoking cessation (*3*). During 2014–2016, an estimated 5.2 million U.S. workers were current e-cigarette users, and prevalence of e-cigarette use was higher among workers in certain industries and occupations (*4*). To estimate recent national prevalence of e-cigarette use among U.S. workers, CDC analyzed 2017–2018 National Health Interview Survey (NHIS) data for adults aged ≥18 years who were employed during the week before the interview. Among an estimated 156 million U.S. workers, 5.3 million (3.4%) were current e-cigarette users (i.e., “every day” or “some days” use), approximately one half of whom also currently used combustible tobacco products. Current e-cigarette use was highest among males, non-Hispanic Whites, those aged 18–24 years, those with no health insurance, those reporting poor or fair physical health, and those who currently used other tobacco products. Prevalence of e-cigarette use was highest among workers in the accommodation and food services industry and in food preparation and serving-related occupations. Continued surveillance of e-cigarette use in the United States, including among workers, is important to inform the development and implementation of evidence-based strategies to minimize population risks of use of e-cigarettes while continuing to explore their potential usefulness for cessation among adult cigarette smokers (*2*,*3*). To maximize the health of workers, employers can integrate comprehensive and effective tobacco cessation programs into workplace health promotion programs (*4**,**5*).

 NHIS is an annual, nationally representative, in-person survey of the noninstitutionalized U.S. civilian population.* The NHIS adult questionnaire is administered to one adult aged ≥18 years randomly selected from each family within the sampled household.^†^ Sample sizes (response rates) for NHIS were 26,742 (53.0%) in 2017 and 25,417 (53.1%) in 2018.^§^

Data analysis included responses from 30,447 adults aged ≥18 years who were “working at a job or business,” “with a job or business but not at work,” or “working, but not for pay, at a family-owned job or business” during the week before the interview. A standardized coding system was used to classify industry of employment and occupation information.^†^ Current e-cigarette users were defined as adults who reported ever using an e-cigarette, even one time, and who reported using e-cigarettes “every day” (daily) or “some days” at the time of the survey. Current e-cigarette use was also assessed by cigarette smoking status (current, former, or never),^¶^ current use of other noncigarette combustible tobacco products (yes or no),** current use of any combustible tobacco products (yes or no),^††^ and current use of any smokeless tobacco products (yes or no).^§§^

Sample weights were adjusted for pooled data to provide nationally representative estimates. Prevalence estimates and 95% confidence intervals were calculated. E-cigarette use was assessed overall for working adults and by sociodemographic characteristics, industry, and occupation. Estimates with a relative standard error ≥30% are not reported. Two-sided t-tests were used to determine statistically significant (p<0.05) differences between point estimates.

During 2017–2018, the prevalence of current e-cigarette use among U.S. workers (3.4%) was significantly higher than that among nonworkers (2.3%). Prevalence was highest among workers who were male (4.1%), non-Hispanic White (4.0%), and aged 18–24 years (7.3%) and among those with a high school education or less (4.7%), with family income <$35,000 (4.9%), with no health insurance (5.0%), and with self-reported poor or fair physical health (5.0%) (Table 1). The prevalence of e-cigarette use was 10.9% among current cigarette smokers, 6.8% among former smokers, 10.4% among users of other combustible tobacco, and 7.3% among smokeless tobacco users. Among the estimated 5.3 million workers who were current e-cigarettes users, 2.3 million (43.1%) were daily e-cigarette users, and 2.6 million (49.5%) also currently smoked combustible tobacco products. Among the estimated 2 million former cigarette smokers, 1.3 million (65.8%) were daily e-cigarette users.

**TABLE 1:** Estimated annual average current electronic cigarette (e-cigarette) use among U.S. workers,* by selected characteristics — National Health Interview Survey, United States, 2017–2018

Characteristic	Estimated no. of workers^†^ (x1,000)	Current e-cigarette users			
		Estimated no. (x1,000)	Prevalence (95% CI)	Daily users, % (95% CI)	Using combustible tobacco,^¶^ % (95% CI)
**Total**	**156,306**	**5,312**	**3.4 (3.1–3.7)**	**43.1 (39.3–46.9)**	**49.5 (45.5–53.4)**
**Age group, yrs**					
18–24	18,781	1,364	7.3 (6.0–8.6)	35.1 (26.5–43.6)	35.8 (27.5–44.1)
25–44	69,089	2,675	3.9 (3.5–4.3)	45.4 (40.4–50.4)	54.0 (48.9–59.0)
45–64	59,267	1,174	2.0 (1.7–2.3)	45.1 (37.9–52.3)	55.6 (48.2–62.9)
≥65	9,169	100	1.1 (0.7–1.5)	67.8 (50.9–84.7)	43.6 (25.3–61.9)
**Sex**					
Male	82,404	3,352	4.1 (3.7–4.5)	46.2 (41.3–51.0)	50.1 (45.1–55.2)
Female	73,902	1,961	2.7 (2.3–3.0)	37.9 (31.8–44.0)	48.3 (41.9–54.7)
**Race/Ethnicity**					
Hispanic	26,629	615	2.3 (1.7–2.9)	28.3 (16.7–39.9)	46.5 (32.9–60.1)
White, non-Hispanic	99,006	3,990	4.0 (3.7–4.4)	45.1 (40.9–49.4)	49.6 (45.4–53.8)
Black, non-Hispanic	18,628	425	2.3 (1.7–2.9)	45.0 (31.0–59.1)	53.5 (37.5–67.8)
Other	12,043	282	2.3 (1.6–3.1)	44.0 (27.1–60.9)	47.9 (30.4–65.3)
**Education**					
≤High school/GED	46,215	2,185	4.7 (4.2–5.3)	45.7 (39.2–52.3)	52.5 (45.8–59.3)
>High school	109,517	3,127	2.9 (2.6–3.1)	41.3 (36.8–45.8)	47.3 (42.6–52.1)
Unknown	574	—**	—	—	—
**Family income**					
<$35,000	26,002	1,267	4.9 (4.2–5.6)	41.2 (34.5–48.0)	54.6 (47.8–61.3)
$35,000–$74,999	41,606	1,602	3.9 (3.3–4.4)	47.5 (41.0–54.1)	54.3 (47.9–60.8)
≥$75,000	76,734	2,089	2.7 (2.4–3.1)	44.0 (37.4–50.7)	45.2 (38.6–51.7)
Unknown	11,964	355	3.0 (1.7–4.2)	24.4 (9.2–39.6)	34.8 (16.1–53.5)
**Health insurance status**					
Not insured	17,206	859	5.0 (4.1–6.0)	42.2 (33.5–50.9)	55.3 (45.7–64.9)
Insured	138,201	4,379	3.2 (2.9–3.4)	43.7 (39.6–47.9)	48.2 (43.8–52.6)
Unknown	899	—	—	—	—
**U.S. Census region^††^**					
Northeast	28,150	658	2.3 (1.8–2.8)	48.4 (38.0–58.7)	50.8 (41.2–60.4)
Midwest	35,277	1,344	3.8 (3.3–4.4)	43.7 (36.0–51.4)	53.0 (46.3–59.7)
South	55,574	2,084	3.8 (3.3–4.2)	42.4 (36.8–47.9)	51.0 (44.5–57.5)
West	37,306	1,227	3.3 (2.7–3.9)	40.9 (31.9–49.9)	42.2 (33.3–51.2)
**Cigarette smoking status^§§^**					
Current	21,040	2,286	10.9 (9.8–12.0)	26.6 (19.6–33.6)	—
Former	29,951	2,045	6.8 (6.0–7.7)	65.8 (59.9–71.7)	—
Never	104,866	981	0.9 (0.7–1.1)	12.5 (4.0–21.1)	—
Unknown	449	—	—	—	—
**Other combustible tobacco use** ^¶¶^					
Yes	7,784	813	10.4 (8.7–12.2)	30.2 (21.9–38.4)	—
No	148,097	4,492	3.0 (2.8–3.3)	45.5 (41.4–49.6)	—
Unknown	427	—	—	—	—
Smokeless tobacco use***					
Yes	4,102	299	7.3 (5.2–9.3)	44.3 (30.3–58.4)	5.6 (4.0–7.2)
No	151,784	5,013	3.3 (3.0–3.6)	43.0 (39.1–47.0)	2.5 (2.3–2.8)
Unknown	420	—	—	—	—
**Self-rated health** ^†††^					
Excellent/Very good/Good	147,048	4,850	3.3 (3.0–3.6)	43.2 (39.2–47.2)	48.7 (44.6–52.7)
Poor/Fair	9,223	458	5.0 (3.7–6.2)	42.0 (28.8–55.1)	58.2 (45.8–70.7)
Unknown	35	—	—	—	—

**Abbreviations:** CI = confidence interval; e-cigarettes = electronic cigarettes; GED = General Education Development certificate.

* Adults who were “working at a job or business,” “with a job or business but not at work,” or “working, but not for pay, at a family-owned job or business” during the week before the interview.

^†^ Weighted to provide national annual average population estimates for current employment.

^§^ Used e-cigarettes at least once during their lifetime and used e-cigarettes “every day” or “some days” at the time of the survey.

**^¶^** Combustible tobacco users were defined as persons who used either “every day” or “some days” at least one combustible tobacco product: cigarettes, cigars, cigarillos, filtered little cigars, pipes, water pipes, or hookahs (for cigarettes, users were defined as persons who had smoked ≥100 cigarettes during their lifetime and smoked “every day” or “some days” at the time of the survey).

** Small sample sizes or prevalence estimates with a relative standard error ≥30% are not presented.

^††^ *Northeast:* Connecticut, Maine, Massachusetts, New Hampshire, New Jersey, New York, Pennsylvania, Rhode Island, and Vermont; *Midwest:* Illinois, Indiana, Iowa, Kansas, Michigan, Minnesota, Missouri, Nebraska, North Dakota, Ohio, South Dakota, and Wisconsin; *South:* Alabama, Arkansas, Delaware, District of Columbia, Florida, Georgia, Kentucky, Louisiana, Maryland, Mississippi, North Carolina, Oklahoma, South Carolina, Tennessee, Texas, Virginia, and West Virginia; *West:* Alaska, Arizona, California, Colorado, Hawaii, Idaho, Montana, Nevada, New Mexico, Oregon, Utah, Washington, and Wyoming.

^§§^ Cigarette smokers were defined as persons who reported smoking ≥100 cigarettes during their lifetime and who currently smoke every day or some days.

^¶¶^ Cigars, cigarillos, or little filtered cigars, or smoked tobacco in a regular pipe, water pipe, or hookah at least once during their lifetime and now smoked at least one of these products “every day” or “some days.”

*** Using chewing tobacco, snuff, dip, snus, or dissolvable tobacco at least once during their lifetime and now used at least one of these products “every day” or “some days.”

^†††^ Perceived self-reported health categorized on the basis of the response to the question “Would you say your health in general is excellent, good, fair, or poor?”

Among the industries assessed, the prevalence of current e-cigarette use ranged from 6.9% among accommodation and food services workers (36.9% were daily users; 49.0% were current combustible tobacco product users) to 1.4% among education services workers (40.0% were daily users; 38.8% were current combustible tobacco product users). Among the occupations assessed, current e-cigarette use prevalence ranged from 7.3% among food preparation and serving-related workers (31.0% were daily users; 47.5% were current combustible tobacco product users) to 1.4% among education, training, and library workers (44.2% were daily users; 29.1% were current combustible tobacco product users). Daily e-cigarette use was highest among workers in the wholesale trade industry and production occupations. Among e-cigarette users, the prevalence of current combustible tobacco product use was highest among workers in the other services industry (including repair and maintenance, private household, and laundry services^¶¶^) and transportation and material moving occupations (Table 2).

**TABLE 2:** Estimated annual average current electronic cigarette (e-cigarette) use among workers,* by industry and occupation — National Health Interview Survey, United States, 2017–2018

Industry/Occupation	Estimated no. of workers (x1,000)	Current e-cigarette users^†^			
		Estimated no. (x1,000)	Prevalence, % (95% CI)	Daily users, % (95% CI)	Using combustible tobacco,^§^ % (95% CI)
**Industry group**					
Accommodation and food services	9,737	669	6.9 (5.0–8.7)	36.9 (24.8–49.0)	49.0 (35.2–62.9)
Transportation and warehousing	6,950	367	5.3 (3.7–6.9)	47.8 (34.6–61.0)	52.1 (37.0–67.3)
Retail trade	15,161	749	5.0 (4.0–5.9)	35.5 (25.8–45.2)	55.0 (45.9–64.2)
Administrative and support, waste management, and remediation services	6,499	297	4.6 (3.3–5.9)	34.9 (20.7–49.0)	52.2 (37.4–66.9)
Information	2,774	124	4.5 (2.0–6.9)	—^¶^	53.5 (25.4–81.5)
Construction	10,996	455	4.1 (3.1–5.2)	48.9 (35.3–62.4)	54.6 (40.5–68.6)
Manufacturing	14,871	510	3.4 (2.6–4.2)	55.9 (44.2–67.6)	48.3 (37.2–59.5)
Real estate and rental and leasing	3,394	113	3.3 (1.8–4.9)	—	38.6 (16.6–60.7)
Public administration	7,807	254	3.3 (2.2–4.3)	50.1 (32.3–67.8)	29.3 (15.4–43.3)
Other services (except public administration)	7,466	243	3.3 (2.0–4.5)	42.8 (24.3–61.2)	62.8 (45.0–80.6)
Wholesale trade	3,574	115	3.2 (1.5–4.9)	79.2 (61.0–97.4)	62.5 (37.2–87.7)
Arts, entertainment, and recreation	3,139	97	3.1 (1.4–4.8)	—	47.6 (18.8–76.4)
Finance and insurance	7,205	215	3.0 (1.6–4.4)	49.1 (30.4–67.7)	—
Health care and social assistance	22,567	478	2.1 (1.6–2.6)	42.5 (31.2–53.8)	51.8 (40.5–63.1)
Professional, scientific, and technical services	13,105	266	2.0 (1.4–2.6)	45.3 (30.6–60.0)	50.0 (34.9–65.0)
Education services	14,612	204	1.4 (0.9–1.9)	39.8 (22.7–56.8)	38.8 (22.0–55.6)
All others**	4,365	131	3.0 (1.6–4.4)	—	—
Refused, not ascertained, don’t know	2,084	—	—	—	—
**Occupation group**					
Food preparation and serving related	7,689	556	7.3 (5.1–9.5)	31.0 (18.2–43.8)	47.5 (31.8–63.1)
Transportation and material moving	9,134	517	5.7 (4.2–7.2)	47.4 (35.2–59.6)	66.1 (54.4–77.8)
Protective service	3,287	169	5.1 (2.8–7.5)	53.3 (29.2–77.4)	50.2 (26.1–74.3)
Sales and related	13,975	667	4.8 (3.8–5.8)	36.9 (26.7–47.2)	50.8 (40.0–61.7)
Installation, maintenance, and repair	4,606	215	4.7 (3.0–6.3)	43.0 (26.2–59.9)	56.6 (39.5–73.7)
Construction and extraction	8,241	353	4.3 (3.0–5.6)	52.2 (36.1–68.3)	49.3 (33.3–65.3)
Production	8,112	341	4.2 (3.0–5.4)	57.4 (44.7–70.2)	39.8 (27.1–52.5)
Arts, design, entertainment, sports, and media	3,709	139	3.8 (2.1–5.4)	45.0 (23.8–66.2)	40.8 (19.7–61.8)
Personal care and service	5,734	184	3.2 (1.8–4.7)	22.7 (3.2–42.3)	56.0 (33.2–78.9)
Business and financial operations	8,959	278	3.1 (1.9–4.3)	48.2 (31.9–64.4)	40.7 (22.4–59.0)
Building and grounds cleaning and maintenance	5,392	161	3.0 (2.0–4.0)	42.6 (25.4–59.8)	48.8 (31.4–66.1)
Office and administrative support	18,875	538	2.9 (2.2–3.5)	46.5 (35.2–57.9)	42.3 (30.6–54.0)
Health care support	3,908	108	2.8 (1.5–4.0)	—	57.1 (33.2–81.0)
Computer and mathematical	5,993	138	2.3 (1.4–3.2)	52.6 (33.1–72.1)	39.1 (20.9–57.4)
Management	15,797	364	2.3 (1.7–2.9)	48.8 (35.9–61.7)	55.4 (42.9–67.8)
Health care practitioners and technical	9,850	160	1.6 (1.1–2.2)	36.4 (19.7–53.0)	45.9 (28.2–63.6)
Education, training, and library	9,614	132	1.4 (0.8–2.0)	44.2 (22.5–65.9)	29.1 (10.7–47.8)
All others^††^	11,540	275	2.4 (1.6–3.2)	—	54.1 (38.2–70.1)
Refused, not ascertained, don’t know	1,890	—	—	—	—

**Abbreviation:** CI = confidence interval.

* Adults who were “working at a job or business”; “with a job or business but not at work”; or “working, but not for pay, at a family-owned job or business” during the week before the interview. Data are weighted to provide national estimates using the survey sample weights for each participant.

^†^ Current users are adults who used e-cigarettes at least once in their lifetime and currently use every day or some days.

^§^ Combustible tobacco use was defined as use either “every day” or “some days” of at least one combustible tobacco product: cigarettes, cigars, cigarillos, filtered little cigars, pipes, water pipes, or hookahs (for cigarettes, users were defined as persons who had smoked ≥100 cigarette during their lifetime and reported currently smoking “every day” or “some days”).

^¶^ Small sample size or prevalence estimates with a relative standard error ≥30% are not presented.

** Includes workers in the agriculture, forestry, fishing, and hunting industry, mining industry, utilities industry, Management of companies and enterprises industry and armed forces industry. Industries with small sample size or ≥30% relative standard error, were combined to improve reliability.

^††^ Includes workers in the architecture and engineering occupation, life, physical, and social science occupation, community and social services occupation, legal occupation, farming, fishing, and forestry occupation, and military occupation. Occupations with small sample size or ≥30% relative standard error were combined to improve reliability.

## Discussion

The prevalence of current e-cigarette use among U.S. workers during 2017–2018 (3.4%) was similar to that during 2014–2016 (3.6%) (*6*). E-cigarette use varied by sociodemographic characteristics, industry, and occupation. Compared with 2014–2016, e-cigarette use prevalence increased among certain subpopulations of workers, especially among young adults. Similar to previous findings, a majority of current adult e-cigarette users reported nondaily use of the products (*7*), and e-cigarette use was associated with use of other tobacco products, mostly notable combustible products (*6*). These findings underscore the importance of continued surveillance of all forms of tobacco products use and the implementation of proven strategies to prevent and reduce tobacco product use among working adults.

Approximately one half of workers who currently use e-cigarettes also smoke combustible tobacco products, with the percentage varying by sociodemographic characteristics, industry, and occupation. Previous findings indicate that many adults reported using e-cigarettes in an attempt to quit smoking (*8*). E-cigarettes have the potential to benefit adult smokers if used as a complete substitute for conventional cigarettes and other combustible tobacco products (*3*). However, e-cigarettes are not approved by the Food and Drug Administration as a smoking cessation aid, and evidence is inadequate to conclude that e-cigarettes, in general, increase smoking cessation (*3*). Moreover, many adult e-cigarette users do not stop smoking cigarettes and instead continue to use both products; in this study, one half of current e-cigarette users also currently smoked combustible tobacco products. Smoking even a few cigarettes per day has health risks (*3*), and the use of cigarettes in combination with e-cigarettes is associated with the same, or in some cases higher, exposure to known tobacco-related toxicants*** compared with using cigarettes alone (*9*). Therefore, adults who use e-cigarettes as an alternative to cigarettes should quit smoking completely rather than use both for an extended period (*3*).

Prevalence of e-cigarette use varied by industry and occupation; prevalence was highest among workers in the accommodation and food services industry and in food preparation and serving-related occupations. Workers in the accommodation and food services industry were generally younger; among those using e-cigarettes, one third used e-cigarettes daily, and approximately one half reported concurrent combustible tobacco product use. Since 2014–2016, e-cigarette use has increased among workers in certain industries, including public administration and in food preparation and serving related, protective services, transportation and material moving, and sales and related occupations (*6*). This increase in e-cigarette use might be attributable, in part, to these industries and occupations having younger workers, less stringent tobacco-free policies, fewer cessation programs, or varying workplace cultures related to tobacco product use (*10*). Implementing targeted workplace interventions that help prevent initiation of tobacco product use and that encourage cessation of all tobacco products among current users can help improve overall worker health.

The findings in this report are subject to at least three limitations. First, only workers employed the week before the interview were included in this study. Some workers might have changed jobs and thus might have been in a different occupation or industry at the time of the survey interview. However, supplementary analyses examining the longest held job yielded similar results. Second, e-cigarette use was self-reported, which could introduce recall bias. Finally, despite data for multiple years being combined, e-cigarette use estimates for some industry and occupation groups were suppressed because of small sample sizes.

Workplace tobacco-control interventions have been effective in reducing cigarette smoking prevalence (*4*). Full implementation of targeted, evidence-based tobacco-control interventions that address the diversity of tobacco products used among U.S. adults, in coordination with regulation of tobacco product manufacturing, marketing, and sales, can reduce tobacco-related disease and death in the United States. To maximize the health of workers, employers can integrate comprehensive and effective tobacco cessation programs (*4*,*5*) into workplace health promotion programs.^†††^

**Summary**

**What is already known about this topic?**

During 2014–2016, an estimated 5.2 million U.S. workers used e-cigarettes, and prevalence was high among certain industries and occupations.

**What is added by this report?**

During 2017–2018, an estimated 5.3 million (3.4%) U.S. workers used e-cigarettes, one half of whom also smoked combustible tobacco products. E-cigarette use was highest among males, non-Hispanic Whites, persons aged 18–24 years, combustible tobacco product users, and workers in the accommodation and food services industry and in food preparation and serving-related occupations.

**What are the implications for public health practice?**

Full implementation of targeted, evidence-based tobacco-control interventions that address the diversity of tobacco products used by U.S. adults, in coordination with regulation of tobacco product manufacturing, marketing, and sales, can reduce tobacco-related disease and death.

## Footnotes

*
https://www.cdc.gov/nchs/nhis/nhis_2018_data_release.htm

†
https://www.cdc.gov/nchs/nhis/data-questionnaires-documentation.htm

§
ftp://ftp.cdc.gov/pub/Health_Statistics/NCHS/Dataset_Documentation/NHIS/2018/srvydesc.pdf

¶ Current cigarette smokers were adults who reported smoking ≥100 cigarettes during their lifetime and who reported smoking “every day” or “some days” at the time of the survey. Former smokers were adults who reported smoking ≥100 cigarettes during their lifetime and reported smoking “not at all” at the time of the survey. Never smokers were adults who reported not having smoked 100 cigarettes during their lifetime.

** Current other combustible tobacco smokers (i.e. no-cigarette combustible tobacco products) were adults who reported ever smoking cigars, little cigars, cigarillos, pipes, water pipes, or hookahs even one time, and currently reported smoking these products “every day” or “some days,” at the time of the survey. Nonsmokers were those who reported never using, or who ever used and reported smoking “not at all” at the time of the survey.

†† Any combustible tobacco users were defined as those who reported current (“everyday” or “some days”) use of cigarettes and/or other combustible tobacco products.

§§ Current smokeless tobacco users were adults who reported ever using smokeless tobacco products that are placed in the mouth or nose (including chewing tobacco, snuff, dip, snus, or dissolvable tobacco) even one time, and reported currently using these products “every day” or “some days” at the time of the survey. Nonusers were those who reported never using, or who ever used and reported using “not at all” at the time of the survey.

¶¶
https://www.bls.gov/iag/tgs/iag81.htm

***
https://www.cdc.gov/workplacehealthpromotion/tools-resources/workplace-health/tobacco-use-cessation.html

†††
https://www.cancer.org/cancer/cancer-causes/tobacco-and-cancer/carcinogens-found-in-tobacco-products.html
